# Progress in Medicine

**Published:** 1896-08-22

**Authors:** 


### Progress in Medicine.

### NEW METHODS OF TREATING ANÆMIA.

A method which in Germany has recently created some attention is that which goes by the name of "the sweat and bleeding cure." Although phlebotomy as a cure for anæmia was recommended by Hoffmann nearly a century ago, and the "sweat cure" is a matter of ancient history, neither separately nor in combination have these methods been seriously considered by the physician of the modern school. Recently, however, a work by Scholz, entitled "Die Behandlung der Bleichsucht mit Schwitz-bädern" (Leipzig, 1890), and another by Wilhelm on "Bleichsucht und Aderlass" (Gustrow, 1890), have aroused interest in these methods of combatting deficiency of hæmoglobin in the blood. Under Paul Schmidt's[1] direction at the Vevein Hospital at Hamburg, these methods have been thoroughly tested and compared with the ordinary methods generally employed. Briefly stated, the results arrived at are as follows: If the body weight and the hæmoglobin contents of the blood be taken as indices with the simple sweat cure (repeated vapour baths) the improvement, if any, was too slight to record. No harmful consequences were, however, observed. The sweat cure combined with blood-letting (eight ounces drawn once, or repeated at intervals) gave questionable results. But the two methods combined with doses of iron gave results which, though good, were no better than when iron alone was administered. The author is of opinion that the advantage which, according to the experience of other observers, follows upon the sweat and blood-letting cure is chiefly due to the rest, nursing, and improved hygienic surroundings which such drastic methods entail. With regard to the iron treatment, Schmidt in all of his observations used Pfeuffer's hæmoglobin pastilles, and strongly recommends them as being more readily absorbed than other forms of iron. In England hæmoglobin is seldom used for this purpose, though hæmatin is sometimes prescribed for the same purpose. Other organic forms of iron, such as the liquor ferri albuminati, and liquor ferri peptonati are better known, and now two other preparations of organic iron combined with Manganese (liquor ferri-mangani saccharati, and liquor ferro-mangani peptonati Dulterich Helfenberg) have been introduced to the notice of the profession in this country. Both of these are said to have a more rapid and noticeable influence on the hæmoglobin contents of the blood than have the simple iron preparation. Other iron preparations recently introduced are worth mentioning in this connection. Ferratin,[2] proposed by Schmiedeberg, is well spoken of by Von Ziemssen and Kündig in doses of 15-30 grains daily, is said to induce a speedy increase in the number of blood corpuscles, and in the amount of hæmoglobin, as well as leading to an increase of body weight. Hæmol and Hæmogallol, proposed by P. Kobert, have been used in Germany and America with some success, and Hæmalbumin, introduced by Dahmer and Finsen,[3] seems to be a durable preparation from blood consisting chiefly of albumins and combined iron One or two teaspoonfuls of the latter may be taken three times daily; it has been tried at several hospitals[4] at Copenhagen with success. The treatment of anæmia by inhalation of ozone has lately been popularised by Labbé and Oudins' paper on the treatment of tuberculosis by the same means. In this paper a convenient form of apparatus is described for generating the gas. A somewhat similar apparatus is now made by the Electric Ozone Syndicate.

[1] Mundrene, Med. Woch., July 9, 1896. [2] Ther. Monalsh, Aug. 14, 1895. [3] Ugeskrift for Lœge, No. 51, 1894. [4] B. M. J. Epit., Feb. 2, 1895.

